# Nitration of chemokine CXCL8 acts as a natural mechanism to limit acute inflammation

**DOI:** 10.1007/s00018-022-04663-x

**Published:** 2023-01-09

**Authors:** Sarah Thompson, Chong Yun Pang, Krishna Mohan Sepuru, Seppe Cambier, Thomas P. Hellyer, Jonathan Scott, A. John Simpson, Paul Proost, John A. Kirby, Krishna Rajarathnam, Neil S. Sheerin, Simi Ali

**Affiliations:** 1 Immunity and Inflammation Theme, Faculty of Medical Sciences, Translational and Clinical Research Institute, Newcastle University, Newcastle upon Tyne, NE2 4HH UK; 2 Department of Biochemistry and Molecular Biology, Sealy Center for Structural Biology and Molecular Biophysics, The University of Texas Medical Branch, Galveston, TX 77555 USA; 3 Department of Microbiology, Immunology and Transplantation, Rega Institute, KU Leuven, 3000 Leuven, Belgium; 4 Department of Respiratory Medicine, Royal Victoria Infirmary, Newcastle Upon Tyne Hospitals NHS Foundation Trust, Newcastle upon Tyne, NE1 4L9 UK; 5 Department of Critical Care Medicine, Royal Victoria Infirmary, Newcastle Upon Tyne Hospitals NHS Foundation Trust, Newcastle Upon Tyne, NE1 4LP UK; 6 Institute for Human Infections and Immunity, The University of Texas Medical Branch, Galveston, TX 77555 USA

**Keywords:** Chemokines, CXCL8, Nitration, Neutrophils, Inflammation, Chemotaxis

## Abstract

Chemokine CXCL8 is a key facilitator of the human host immune response, mediating neutrophil migration, and activation at the site of infection and injury. The oxidative burst is an important effector mechanism which leads to the generation of reactive nitrogen species (RNS), including peroxynitrite. The current study was performed to determine the potential for nitration to alter the biological properties of CXCL8 and its detection in human disease. Here, we show peroxynitrite nitrates CXCL8 and thereby regulates neutrophil migration and activation. The nitrated chemokine was unable to induce transendothelial neutrophil migration in vitro and failed to promote leukocyte recruitment in vivo. This reduced activity is due to impairment in both G protein-coupled receptor signaling and glycosaminoglycan binding. Using a novel antibody, nitrated CXCL8 was detected in bronchoalveolar lavage samples from patients with pneumonia. These findings were validated by mass spectrometry. Our results provide the first direct evidence of chemokine nitration in human pathophysiology and suggest a natural mechanism that limits acute inflammation.

**Supplementary Information:**

The online version contains supplementary material available at 10.1007/s00018-022-04663-x.

## Introduction

Neutrophils form an essential component of the innate immune response. They mediate their anti-pathogenic and repair functions [1] via a series of mechanisms, including releasing anti-microbial proteins and enzymes [2], degranulation and oxidative burst involving rapid production of reactive oxygen species (ROS) and reactive nitrogen species [3, 4]. These potent mechanisms, while effective in clearing pathogens, act as a double-edged sword; it causes susceptibility to infection when neutrophil function is deficient, and excessive neutrophil activation that is detrimental, as is the case in conditions such as reperfusion injury, organ rejection, and acute respiratory distress syndrome (ARDS) [2, 5]. Consequently, neutrophils can damage healthy tissue and exacerbate inflammation.

As one of the key neutrophil-recruiting chemokines, CXCL8 is produced and released by a wide variety of cell types in response to a range of stimuli including microbial products, injury, and hypoxia [6–8]. CXCL8 production is regulated by gene transcription and translation, but function is also determined by whether CXCL8 exists as monomers or dimers, its interaction with receptors (CXCR1 or CXCR2), downstream signaling pathways and binding to sulfated glycosaminoglycans (GAG) [9, 10]. CXCL8 can also be regulated by atypical chemokine receptor 1 (ACKRI) which scavenges circulating CXCL8 and is generally thought to have an anti-inflammatory function [11].

Recently, various post-translationally modified forms of CXCL8 were resolved in the synovial fluid of patients with arthritis using high resolution mass spectrometry [12]. Several chemokines including CCL2 [13] CCL5 [14] and CXCL12 [15] have been shown to be modified by peroxynitrite, a reactive species produced by neutrophils, which can post-translationally nitrate amino acids such as tyrosine and tryptophan, and influence chemokine function [16]. Peroxynitrite, a product of the reaction between nitric oxide and the superoxide anion (O_2_^−^), has a short half-life of 1.9 s, and so can only modify proteins in its immediate proximity (~ 20 µm range)[17]. Peroxynitrite nitrates tyrosine residues to form 3-nitrotyrosine (3-NT), which is a marker of nitrative stress, and has been documented in several human diseases. Although in vitro nitration of several chemokines has been described, unambiguous ex vivo or in vivo detection has been challenging due to a lack of sensitive analytical, biochemical and chemical tools and probes.

In this study, we produced nitrated CXCL8 by treating recombinant CXCL8 with peroxynitrite and used this as a target to generate highly specific antibodies for detecting nitrated CXCL8. Using cellular assays, animal models, biophysical and structural studies, we show that nitration of CXCL8 impairs neutrophil chemotaxis, receptor signaling and GAG binding. We also show nitrated CXCL8 in bronchoalveolar lavage (BAL) samples from patients with suspected ventilator-associated pneumonia (VAP). We conclude that nitration may function as a feedback mechanism to dampen CXCL8 activity.

## Materials and methods

All experiments and laboratory work were carried out according to the Control of Substances Hazardous to Health (COSHH) and BioCOSHH regulations, and according to Newcastle University’s Safety Policy, “Safe Working with Biological Hazards” and “Safe Working with Chemicals in the Laboratory” guidelines. Cell culture work was performed according to the regulations for the containment of class II pathogens. Animal work was approved by the Home Office UK, and carried out under Project Licence number 66/4497, protocol number 19b2/19b4.

### Patient samples

BAL samples from patients with suspected-VAP and healthy controls were collected as part of study approved by a Research Ethics committee (11/NE/0242) [18].

### Chemokine nitration

Recombinant CXCL8 (72 amino acids) at a concentration of 1 mg/ml was incubated with 1 mM peroxynitrite for 15 min at 37 °C, then dialyzed overnight against water. A sample was tested by NMR and MS2 analysis to ensure nitration was successful. The Y13F-CXCL8 mutant was generated according to methods described earlier [10] to examine the role of tyrosine in CXCL8 nitration.

### Generation of nitrated CXCL8 antibody

Bio-Rad developed custom recombinant monoclonal HuCAL^®^ antibody using positive and negative screening of proteins against a phage display library of antibodies. This library consists of human antibody genes synthetically made to cover > 95% of the structural immune repertoire, that are cloned in *E. coli* expression vectors. Panning and screening was performed to find candidate antibodies that would preferentially recognize the nitrated CXCL8 but not wild type CXCL8 in Bio-Rad’s optimized direct ELISA assays. Potential candidate antibodies that display > fivefold higher specificity for nitrated over wild type CXCL8 were produced as bivalent Fab-bacterial alkaline phosphatase fusion antibodies with FLAG^®^ and Histidine 6 tag.

### Human neutrophil isolation

Primary neutrophils were isolated from whole blood of healthy volunteers. Ethical approval to obtain blood from healthy volunteers was granted by the County Durham and Tees Valley Research Ethics Committee (12/NE/0121). Primary neutrophils were isolated by dextran sedimentation (Dextran T500; Pharmacosmos, Holbaek, Denmark) and centrifugation on Percoll (GE Healthcare, Buckinghamshire, UK) density gradients as previously described. Neutrophils were rested for 1 h in serum-free medium (0.1% BSA in RPMI-1640, with 2 mM l-glutamine, 100 U penicillin and 0.1 mg/ml streptomycin) at 37 °C and 5% CO_2_ before being used in experiments.

### Cell culture

The human microvascular endothelial cell line HMEC-1[19] was grown in complete MCDB-131 medium (ThermoFisher Scientific) (10% FBS supplemented with 10 ng/ml epidermal growth factor (EGF), 1 µg/ml hydrocortisone, 2 mM l-glutamine, 100 U penicillin and 0.1 mg/ml streptomycin).

### Chemotaxis assays

Ibidi^®^ μ-slide chemotaxis assays were performed according to Ibidi^®^’s application note 17 (ibidi GmbH, Munich, Germany). 3 × 10^6^ neutrophils were seeded into the central channel, and a gradient of CXCL8 or nitrated CXCL8 in serum-free medium (30 nM highest concentration) was created across the channel. Serum-free medium alone was used as a negative control. Slides were then imaged every 2 min for 3 h using a Nikon TiE Multi-Modality microscope, and images were analyzed using Fiji’s manual tracking plug-in. The tracks of 40 randomly chosen cells were recorded and imported to Ibidi^®^’s Chemotaxis and Migration Tool, where they were analyzed for parameters including velocity, directness, Euclidean distance, and forward migration index.

Trans-filter transwell chemotaxis assays were performed in 24-well companion plates (BD Falcon) using cell culture inserts with 3 µm pores. CXCL8 and nitrated CXCL8 in serum-free medium (0–30 nM) were added to the wells, and 3 × 10^5^ neutrophils were added to the upper chamber of the inserts. The assay was incubated to allow migration for 1.5 h at 37 °C. Neutrophils that had migrated into the lower chamber were counted using CountBright Absolute Counting Beads (LifeTechnologies) as per the manufacturer’s instructions on a FACS Canto II flow cytometer.

Trans-endothelial transwell chemotaxis assays were performed as described above, though 72 h prior to the assay, inserts were seeded with 2 × 10^5^ HMEC-1 cells in 1 ml complete MCDB-131 medium. All assays were performed in triplicate.

### Calcium flux assay

Primary neutrophils were isolated and rested as previously described [20]. Cells were washed in HBSS supplemented with 1 mM CaCl_2_, 1 mM MgCl_2_ and 1% FBS(v/v) and resuspended to 1 × 10^7^ cells/ml. Neutrophils were stained with 3 µM Indo-1 (ThermoFisher Scientific) for 30 min at 37 °C. The stained cells were washed twice in supplemented HBSS by centrifugation at 400×*g* for 5 min and resuspended at 3 × 10^6^ cells with supplemented HBSS before resting in a 37 °C water bath for 30 min. A baseline reading at 420 nm and 510 nm was procured using a Fortessa X20 flow cytometer for 1 min before the addition of treatments (PBS, CXCL8, nitrated CXCL8, or Y13F CXCL8). Fluorescence was recorded for 5 min before the addition of 2 mM ionomycin and further response was recorded for 2 min. Ratio of 420 nm emission to 510 nm emission was calculated using FlowJo v10 software.

### β-Arrestin recruitment assay

β-Arrestin recruitment activity of CXCL8 variants was measured using CXCR1 and CXCR2 PathHunter Kits (DiscoveRx). The assay was carried out according to the manufacturer’s protocol. The transfectants were cultured using the cell plating reagent in 96-well white-walled, clear-bottom plates for 48 h. Chemokine variants over a range of concentrations were added to the cells and incubated for 90 min at 37 °C. The detection reagents were then added and incubated for 1 h in the dark. β-Galactosidase-induced luminescence upon β-arrestin–CXCR1/CXCR2 interaction was then measured using BMG Optima.

### Immunoblotting

For the detection of pERK, primary neutrophils were isolated as described previously [20] and resuspended in serum-free RPMI (2 × 10^6^ cells in 1 ml per treatment tube) and rested for 1 h at 37 °C prior to treatment. Cells were stimulated for 2, 5 or 10 min with 30 nM of CXCL8 or CXCL8 variants and placed immediately on ice. Neutrophils were washed with ice-cold PBS and resuspended in 200 µl of cell lysis buffer (10 ml Cell Lytic M (Sigma) plus 1 × PhosSTOP™ phosphatase inhibitor (Roche) and 1 × cOmplete™ protease inhibitor tablet (Roche) on ice for 10 min with intermittent vortexing. Cell lysate was electrophoretically separated by SDS-PAGE and transferred to nitrocellulose membranes. Membranes were blocked for 1 h at room temperature on 0.1% Tween Tris-buffered saline (TBST) with 5% BSA and probed with anti-human-pERK1/2 antibody (36-8800, ThermoFisher Scientific) at 0.2 µg/ml or serum from a patient with primary biliary cholangitis (containing antibodies against pyruvate dehydrogenase complex 2 (PDC-E2) at 1:5000 dilution). Detection was carried out using anti-rabbit-HRP secondary antibody (1:10,000) or anti-human-HRP antibody (1:5000) for 1 h at RT. Finally, membranes were washed, and bands were visualized by enhanced chemiluminescent substrate (SuperSignal West Pico substrate) and imaged with Fc Odessey (Li-Cor).

Detection of naturally occurring endogenous nitrated CXCL8 in BAL samples involved denaturing 10–50 µg of BAL protein, which was electrophoretically separated by SDS PAGE, and transferred to nitrocellulose membranes. Membranes were blocked for 1 h at room temperature in TBST with 5% fat-free milk or 5% BSA (when using 3-NT or Bio-Rad AbD31649.1 primary antibody) and probed overnight at 4 °C. Rabbit polyclonal anti-CXCL8 antibody (AHC0881, LifeTechnologies) at 1 µg/ml, Bio-Rad HuCAL^®^ antibody (AbD31649.1) at 2 µg/ml and mouse monoclonal anti-3-NT antibody (HM5001, HycultBiotech) at 0.1 µg/ml were used. Detection was carried out using an anti-rabbit-HRP secondary antibody (1:10,000 Sigma) for CXCL8, or mouse monoclonal anti-FLAG secondary antibody (1:2000, Proteintech) followed by anti-mouse secondary antibody (1:10,000, Sigma) for Bio-Rad HuCAL^®^ antibody (AbD31649.1) and anti-mouse secondary antibody (1:10,000, Sigma) for 3-NT, for 1 h at RT. Finally, membranes were washed, and bands were visualized by enhanced chemiluminescent substrate (SuperSignal West Pico substrate) and exposed to X-ray films.

### Immunoprecipitation

Bronchoalveolar lavage samples from patients with suspected-VAP were immunoprecipitated using a commercially available 3-NT IP kit (Cayman Chemical) following the manufacturer’s protocol with the modification of changing wash buffer to phosphate buffered saline (PBS). Protein pulldown was subsequently subjected to immunoblotting as stated with rabbit anti-CXCL8 polyclonal antibody.

### Murine intraperitoneal recruitment

Eight-week-old female BALB/c mice (Charles River, UK) were injected intraperitoneally with 500 µl of PBS ± 1 µg CXCL8 or nitrated CXCL8. Six hours later mice were culled by cervical dislocation under anesthesia and the abdominal cavity was lavaged three times with 1 ml PBS, 3 mM EDTA. Lavage samples were centrifuged at 500*g* for 5 min and resuspended in 125 µl PBS buffer. A TALI image-based cytometer (Thermo Fisher Scientific) was used to estimate total cell counts on 25 µl of sample while the remaining 100 µl was stained with fluorescently-conjugated antibodies (against CD45, CD14 and Ly6G) and analyzed using a FACS Canto II flow cytometer. Samples were analyzed using FlowJo v10 software.

### Surface plasmon resonance

Surface plasmon resonance (SPR) studies were performed using a BIAcore3000 as described previously [20]. Briefly, heparin was biotinylated at the reducing end and immobilized to a SA sensor chip (pre-coated with streptavidin, GE Healthcare). The chip surface was activated with 50 μl 0.2 M 1-ethyl-3-(3-dimethylaminopropyl)-carbodiimide and 50 μl 0.05 M *N*-hydroxysuccinimide before injection of 50 μl of streptavidin (0.2 mg/ml in 10 mM acetate buffer, pH 4.2). Remaining activated groups were blocked with 1 M ethanolamine HCl, pH 8.5 for 5 min. Biotinylated heparin was immobilized by injecting 5 μl of 50 μg/ml in Hanks’ Balanced Salts with 0.3 M NaCl at a flow rate of 5 μl/min and injections repeated until a resonance unit (RU) increase of 200 RU was reached after which the surface was washed with 2 M NaCl. For binding assays, a range of chemokine concentrations (5–1000 nM) were passed across the chip at 30 μl/min for 2 min followed by a 300 s dissociation phase and a 2 min injection of 1 M NaCl to regenerate the sensor surface. RU from a flow cell coated with streptavidin only was subtracted from the results from heparin-coated flow cells and analysis was performed using BIAevaluation 4.1 software.

### Statistical analyses

All results are expressed as means ± SEM of replicate samples. The statistical significance of changes was assessed by the application of one-way ANOVA with Dunnett’s or Tukey’s post-test as appropriate. All data were analyzed using Prism 7.1 software.

## Results

### Generation of characterization of nitrated CXCL8

To study the function of nitrated CXCL8 and to develop an antibody that is specific for nitrated CXCL8, we optimized a production protocol by using peroxynitrite to nitrate recombinant CXCL8. Ion trap MS2 analysis of nitrated CXCL8 was performed to determine the consequence of modification by peroxynitrite. The most abundant peak (1205) was isolated, retained within the ion trap, then fractionated at the D–P bond to create an N-terminal fragment (1493.0), and a C-terminal fragment (820.1) as shown in Supplementary Fig. 1. This figure demonstrates how these fragments were analyzed, using the ion mass and the charge to calculate the molecular weight of each fragment. The C-terminal fragments of both the wild type CXCL8 and nitrated CXCL8 had the same *m*/*z* and therefore same molecular mass, but the N-terminal fragment of the nitrated chemokine was found to have a molecular mass 45 Da larger than that of the wild type chemokine. This suggests that the only tyrosine (Y13) present within the N terminal half of the chemokine is the nitration site. Nitration of the two histidines (H18 and H33) was excluded by NMR as discussed below.

**Fig. 1:**
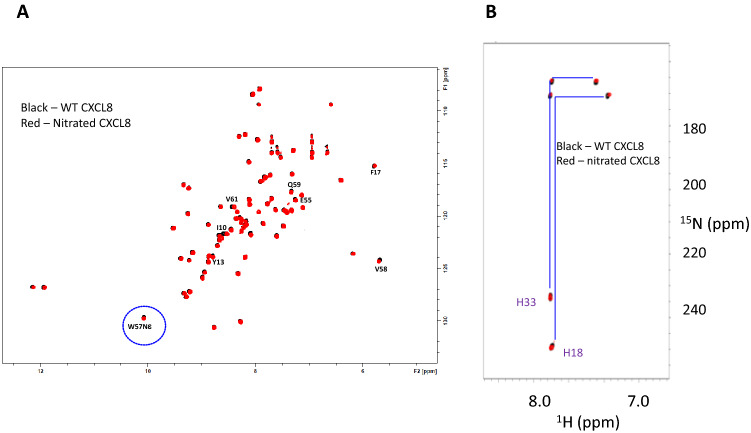
NMR analysis of wild type and nitrated CXCL8. **a** ^1^H–^15^N-HSQC spectra of wild type CXCL8 and nitrated CXCL8 are essentially identical, indicating tyrosine nitration has minimal impact on global fold and overall tertiary structure. Similar chemical shifts of the W57NƹH between wild type and nitrated CXCL8 indicate that the only tryptophan remains unmodified post-peroxynitrite treatment. **b** The histidine side chain imidazole chemical shifts indicated by ^1^H–^15^N-HMQC spectra between CXCL8 and nitrated CXCL8 are identical, indicating both the histidine residues are not modified

Knowledge of how nitration impacts the tertiary structure is necessary to establish that any changes in binding interaction and function is due to nitration and not due to gross structural changes. We characterized the nitrated CXCL8 by solution nuclear magnetic resonance (NMR) spectroscopy. NMR chemical shifts are exquisitely sensitive to secondary and tertiary structures, and therefore, chemical shifts can not only validate structural integrity but also identify which residues are modified/perturbed as a consequence of nitration. ^1^H–^15^N heteronuclear single quantum coherence (HSQC) NMR spectrum of nitrated CXCL8 was essentially the same as the unmodified CXCL8 [10], indicating that nitration has minimal impact on the overall tertiary structure and fold (Fig. 1a). The chemical shifts of the indole side chain NƹH of the only tryptophan (circled in blue) is similar for nitrated and native CXCL8 indicating that the tryptophan is not modified in the nitrated protein. We also measured the NMR spectrum of histidine side chain (Fig. 1b), and observe that the chemical shifts of both histidines (H18 and H33) in nitrated CXCL8 is the same as in the wild type CXCL8 [21]. The NMR data collectively indicates that nitration is restricted to a single tyrosine and that the functional phenotype of the nitrated chemokine can be confidently attributed to the modification and not due to any other structural changes.

### Activity of nitrated CXCL8 for neutrophil migration in vivo

**Fig. 2:**
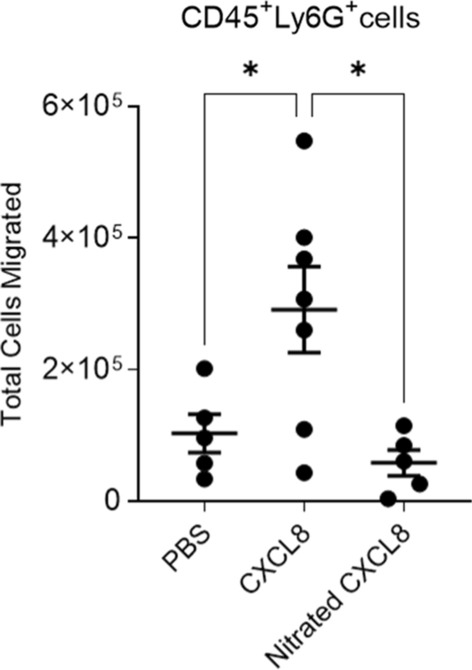
Murine intra-peritoneal recruitment in response to wild type or nitrated CXCL8. Total neutrophil migration into the mouse peritoneum were measured 6 h after intra-peritoneal administration of PBS, 1 μg wild type CXCL8 or 1 μg nitrated CXCL8. Shown are the total numbers of live neutrophils (Sytox Red—CD45+ Ly6G+). Each data point represents an individual mouse. Statistical analysis was performed using a one-way ANOVA with Tukey’s post-test. *p < 0.05

To determine the impact of nitration on in vivo CXCL8 function, variants of CXCL8 were injected into the peritoneum of mice and leukocytes were allowed to migrate for 6 h. Despite the lack of a gene coding for CXCL8, mice possess a receptor homologous to human CXCR2 that are able to mediate neutrophil chemotaxis in response to human CXCL8 [22]. Cells were collected by lavage and the infiltrating leukocytes were characterized and quantified (gating strategy shown in Supplementary Fig. 2). Neutrophil migration in response to nitrated CXCL8 was significantly reduced compared to wild type CXCL8 (Fig. 2) and was no different compared to the control group.

To investigate the potential of the nitrated CXCL8 to antagonize the inflammatory activity of wild type CXCL8, a series of murine air pouch experiments were performed. Air pouches were formed by s.c. injection of air into the back of mice. Injection of 1 µg of wild type CXCL8 induced significant migration of neutrophils, whereas migration in response to nitrated CXCL8 was significantly reduced. A 1:1 mixture of CXCL8 and nitrated resulted in recruitment of neutrophils, which was not significantly different to CXCL8 alone (Supplementary Fig. 3). These data suggest that nitrated CXCL8 in not able to compete with the wild type CXCL8 to inhibit localized CXCL8 mediated inflammation in the air pouch.

### Activity of nitrated CXCL8 for neutrophil migration in vitro

**Fig. 3:**
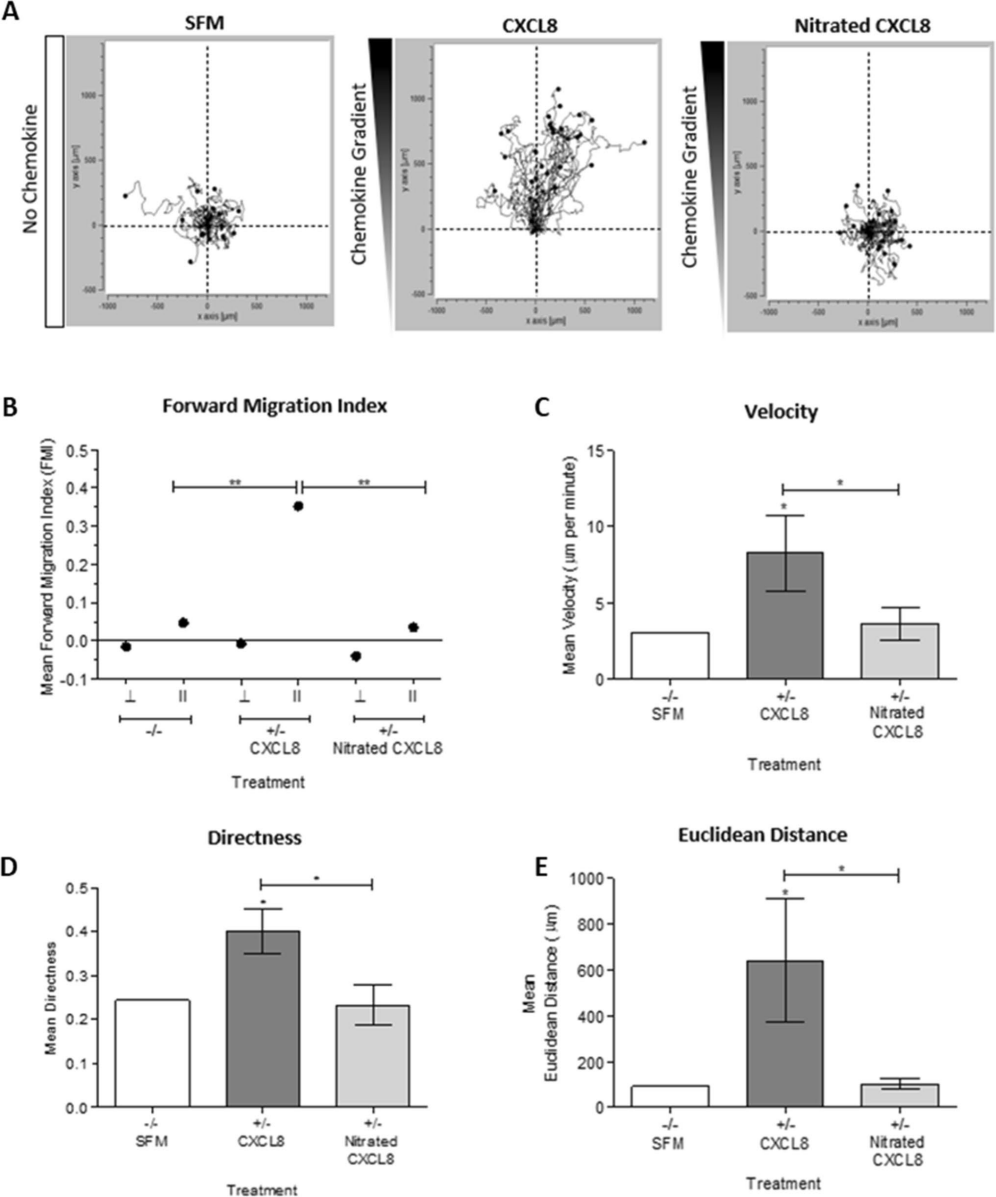
Time-lapse microscopic analysis of neutrophil migration towards CXCL8 or nitrated CXCL8. Neutrophil chemotaxis towards a gradient of either wild type CXCL8 or nitrated CXCL8 was assessed by live cell tracking using Ibidi µ-Chemotaxis Slides. Neutrophil migration along a chemotactic gradient created using serum-free media (SFM) and either 30 nM wild type or nitrated CXCL8 (no gradient using only SFM was also used as a negative control) were observed under a Nikon Multi-Modality inverted microscope. Image analysis was performed using FIJI’s manual tracking plugin with the Chemotaxis and Migration Tool. **a** Trajectory animations showing neutrophil chemotaxis in response to SFM, 30 nM wild type CXCL8 or 30 nM nitrated CXCL8. **b** Mean forward migration index [perpendicular (⊥) and parallel (ll) to the direction of the chemokine gradient], **c** velocity, **d** directness and **e** Euclidean distance of the cells were analyzed. Data shown in trajectory animation are representative of three independent experiments. Trajectory analysis is combined from three independent experiments, each with one technical replicate and neutrophils isolated from different blood donors. Statistical analysis was performed using a One-Way ANOVA with Tukey’s post-test. *p < 0.05; **p < 0.01

**Fig. 4:**
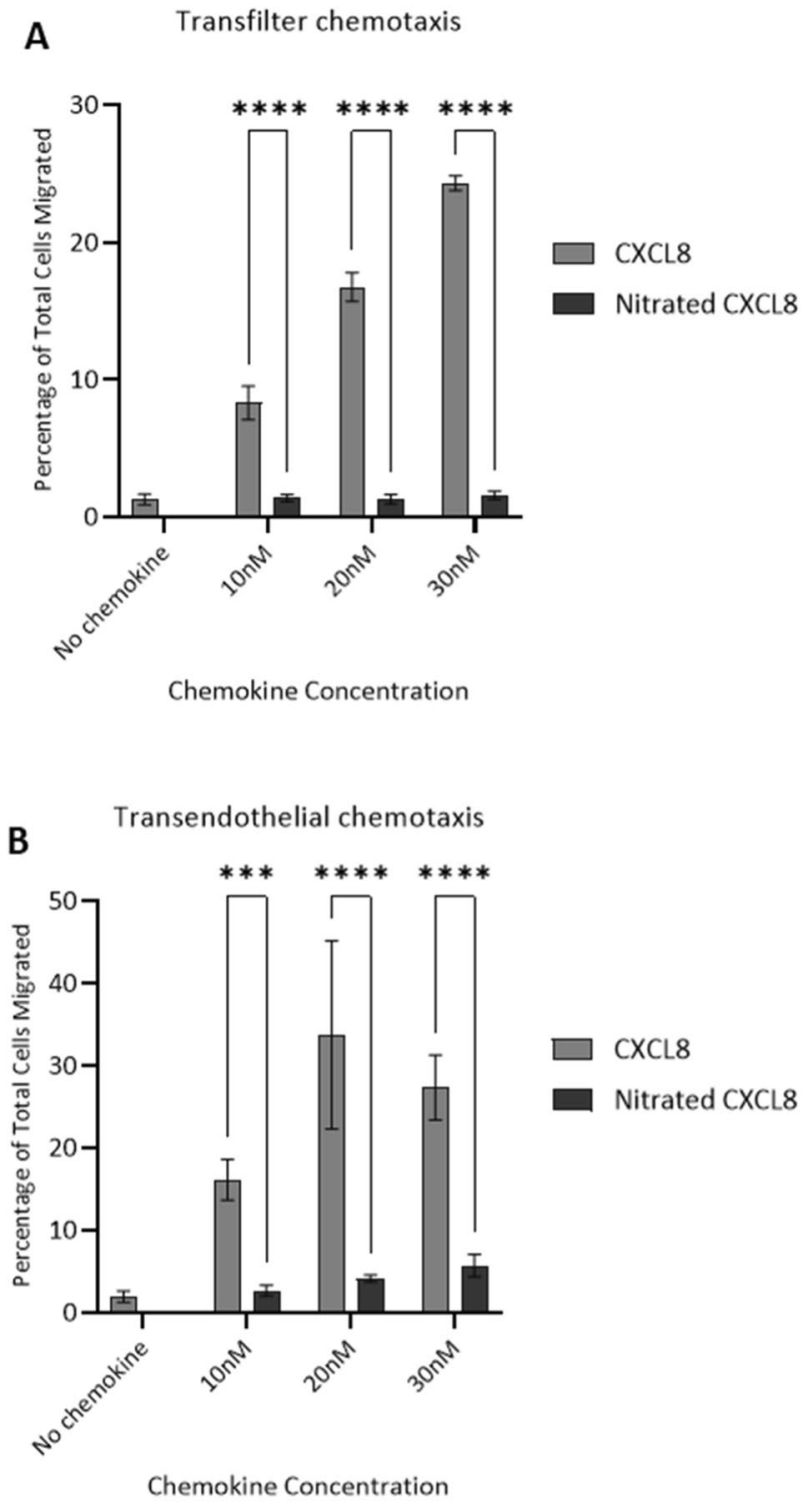
Ability of wild type CXCL8 and nitrated CXCL8 to induce neutrophil migration in vitro. **a** Trans-filter chemotaxis assays showing percentage of total neutrophils that migrated through a bare filter in response to 30 nM, 20 nM and 10 nM of wild type CXCL8 and nitrated CXCL8. **b** Trans-endothelial chemotaxis assays showing percentage of total neutrophils that migrated through an HMEC-1 endothelial cell monolayer in response to the above concentrations of CXCL8 variants. Data shown in both **a**, **b** are representative of two independent experiments, each with three technical replicates and neutrophils isolated from different blood donors. Statistical analysis was performed using one-way ANOVA with Šídák post-hoc test. ***p ≤ 0.001, ****p ≤ 0.0001

As the in vivo recruitment of neutrophils requires receptor and GAG binding, we investigated these interactions for nitrated CXCL8 using in vitro assays. Neutrophil migration was investigated using live cell tracking. CXCL8 induced directed neutrophil migration along the chemokine gradient compared to a random pattern in the control, serum-free medium (Fig. 3). Nitrated CXCL8 was highly impaired in its activity in terms of speed, direction, distance travelled, and forward migration index compared to wild type CXCL8, and its profile was no different from that of the control. We next determined the effect that nitration had on the ability of CXCL8 to induce neutrophil chemotaxis in a trans-filter chemotaxis assay. The results showed that CXCL8 induces significant neutrophil chemotaxis in a concentration-dependent manner whereas nitration abrogated chemotactic function at all concentrations tested (Fig. 4a).

We also carried out trans-endothelial chemotaxis assays with neutrophils migrating through a confluent monolayer of HMEC-1 cells grown onto the filter in response to the matching concentrations of CXCL8 and nitrated CXCL8. This experimental setup mimics neutrophils infiltrating the tissue by migrating through the wall of the blood vessel. The nitrated chemokine was essentially inactive, similar to observations in the acellular trans-filter chemotaxis assay (Fig. 4b).

### Interaction of nitrated CXCL8 with glycosaminoglycans

**Fig. 5:**
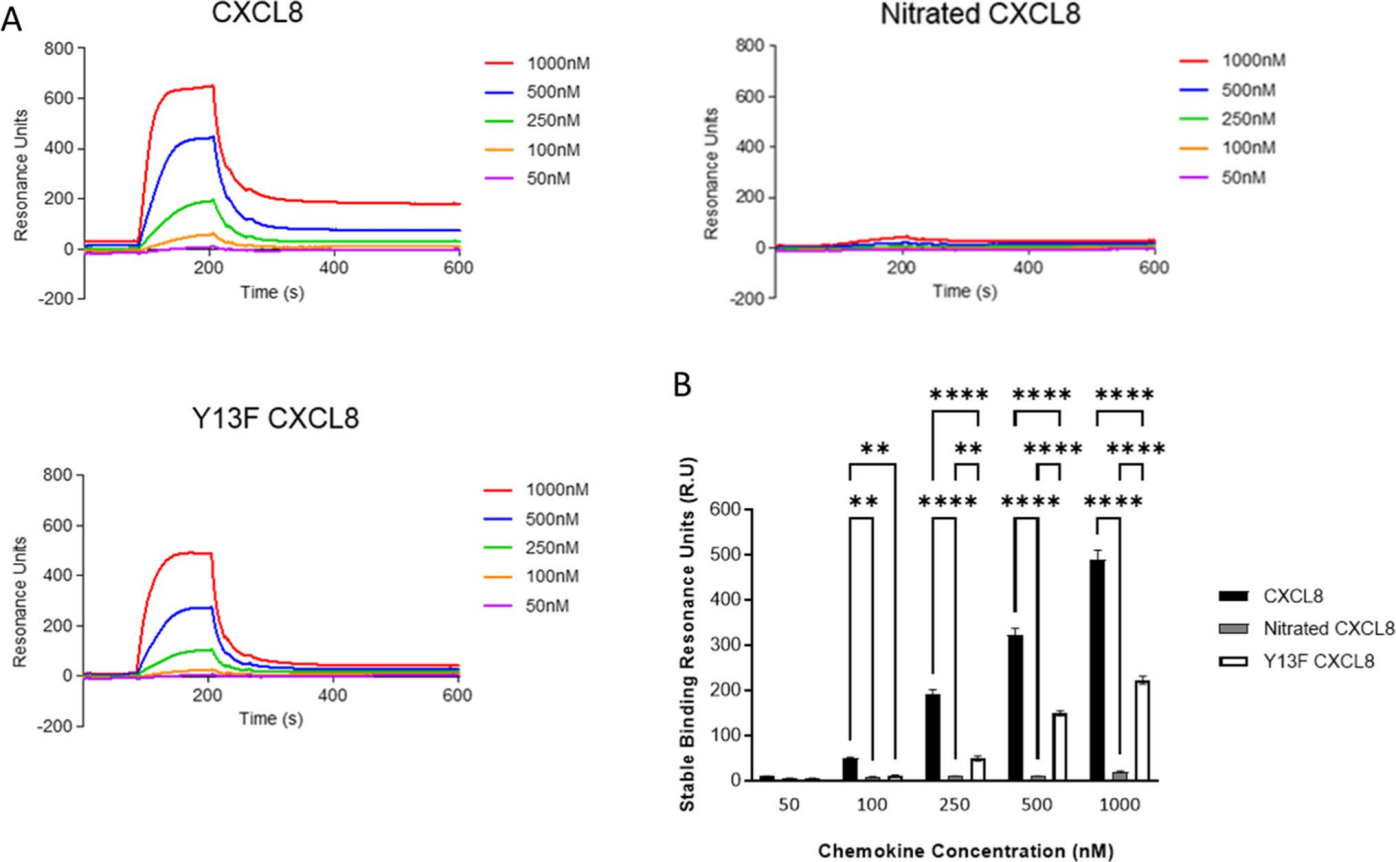
Heparin binding activity of CXCL8 variants. **a** Assessment of heparin binding of wild type CXCL8, nitrated CXCL8 and Y13F CXCL8 was carried out using Biacore. **b** Stable binding points of wild type CXCL8, nitrated CXCL8 and Y13F mutant CXCL8 as reported by Biacore software. 50–1000 nM chemokine was flowed over immobilized heparin (159.6RU bound) and the alteration in response units (RU) shown. Data shown in **a** are combined from, and in **b** are representative of, three independent experiments, each with one technical replicate. Statistical analysis was performed using a one-Way ANOVA with Tukey’s post-test. **p ≤ 0.01; ***p ≤ 0.001

CXCL8 activity is regulated by binding to GAGs [23–25]. We used SPR to characterize the impact of nitration on binding to heparin, which is widely used as a surrogate to mimic binding to endothelial and glycocalyx heparan sulphate, under flow conditions. In this assay, we also used the Y13F–CXCL8 mutant, in which the tyrosine was mutated to phenylalanine, to probe the importance of tyrosine for chemokine function. We designed the Y13F mutant as a control to better describe the functional results of the nitrated CXCL8. Compared to adding a bulky nitro group, mutating the hydroxyl group to a proton results in less perturbation to the local and global structure and hence function. Indeed, our data show that the Y13F mutation impairs trans-endothelial chemotaxis but is less detrimental compared to nitration (Supplementary Fig. 4A). Results show that nitration of CXCL8 almost completely abolishes the ability to bind heparin—stable binding at 1000 nM were 480 RU CXCL8, compared to 26 RU for nitrated CXCL8. Y13F-CXCL8 mutant shows a smaller decrease in heparin binding (241 RU) compared to nitrated CXCL8 (Fig. 5).

Heparan sulfate (HS) is intrinsically heterogeneous due to non-template driven sulfation and a modular structure consisting of sulfated and non-sulfated domains. We and others have shown that chemokines bind to the sulphated domain. Our recent NMR studies for related chemokines CXCL1 and CXCL5 binding to commercial heparan sulfate and heparin polymers show that the same amino acids engage both GAGs [26]. The observation that nitration abrogates heparin binding suggests that nitrated CXCL8 will also be impaired for heparan sulfate binding. We show that is the case by measuring binding of wild type CXCL8 and nitrated CXCL8 to heparan sulfate using plate assays. The binding affinity of nitrated CXCL8 was significantly reduced compared to wild type CXCL8 (Supplementary Fig. 4B).

### Activity of nitrated CXCL8 for receptor signaling

**Fig. 6:**
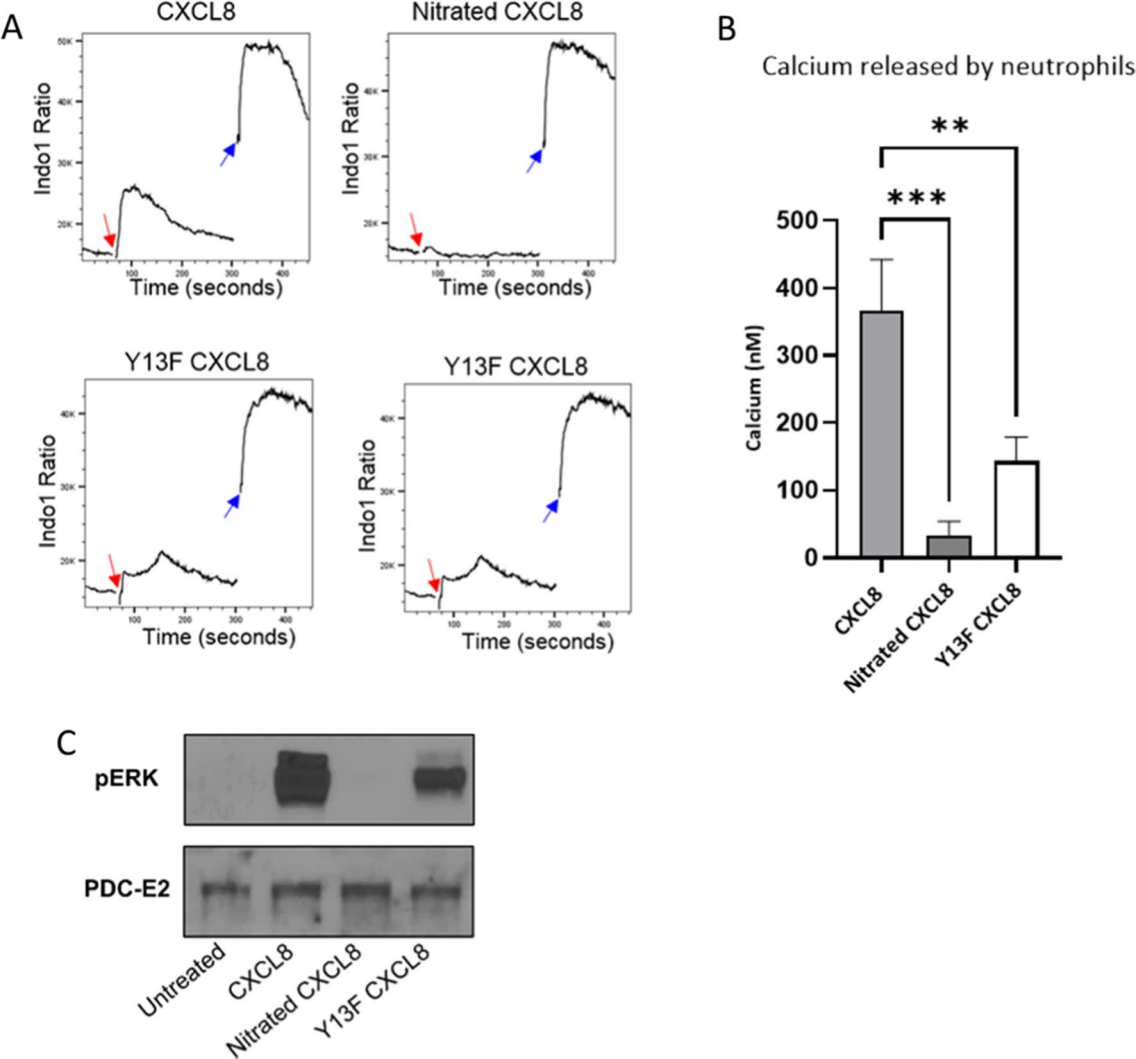
Effect of CXCL8 nitration on neutrophil signaling. **a** Neutrophil calcium flux in response to exposure of CXCL8 variants after 60 s (red arrow).10 µg/ml of ionomycin was used as a positive control and added at 300 s (blue arrow). **b** Intracellular calcium mobilization (nM) in response to PBS or 30 nM of CXCL8, nitrated CXCL8 and Y13F CXCL8 was calculated using the equation [Ca^2+^] = Kd × (*R* − *R*_min_)/(*R*_max_ − *R*), where the Kd = 844 nM/l. **c** Neutrophils were treated for 5 min with 30 nM of the chemokine variants. Phosphorylation of ERK was assessed using western blot with a pERK1/2 antibody and an anti-pyruvate dehydrogenase complex (PDC-E2) antibody as loading control. Data shown in **a**, **c** are representative of three independent experiments, each with one technical replicate, and neutrophils isolated from different blood donors. Statistical analysis was performed using a one-way ANOVA with Tukey’s post-test. *p < 0.05; **p < 0.01; ***p < 0.001

**Fig. 7:**
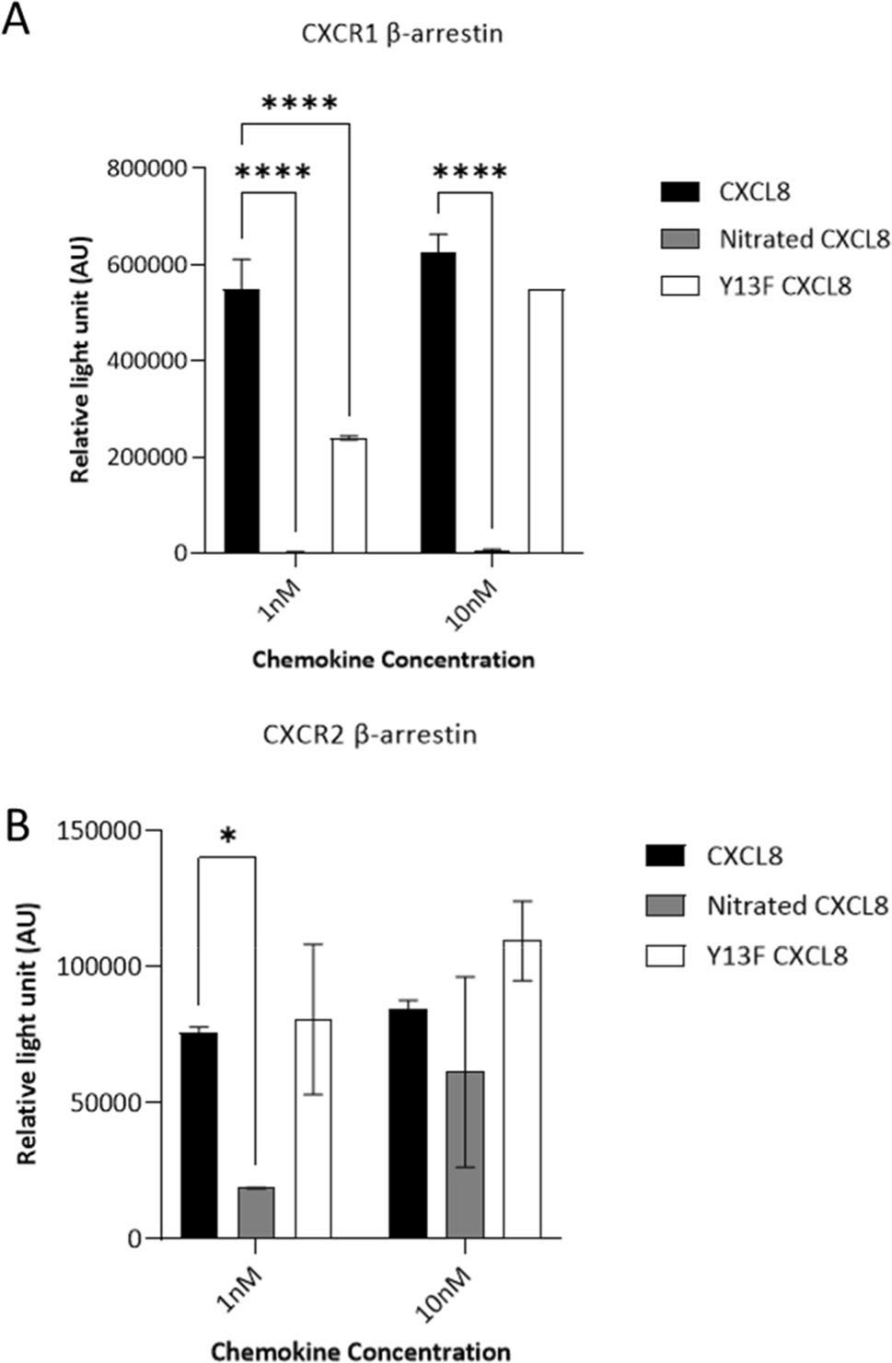
β-Arrestin recruitment activity of CXCL8 variants. Chemokine variants were added at 1 or 10 nM concentration to the transfected cells and β-galactosidase-induced luminescence upon β-arrestin–CXCR1/2 interaction was measured. Data were collected in triplicate, and the results are expressed as mean ± SD, and are representative of *n* = 3. Statistical analysis was performed using two-way ANOVA with Dunnett’s multiple comparison. *p < 0.05, ****p < 0.0001

CXCL8 is a potent agonist for CXCR1 and CXCR2 receptors, and activation of both receptors triggers G protein and β-arrestin signaling pathways [27–29]. We aimed to assess whether nitration of CXCL8 impairs receptor-dependent neutrophil migration. We used calcium flux and ERK phosphorylation in neutrophils as readouts to quantify G protein signaling. CXCL8 induced a calcium flux of ~ 350 nM above the control level, whereas nitrated CXCL8 showed tenfold lower activity. Lower activity of the Y13F CXCL8 also indicated that the Tyr13 residue plays an important role in CXCR1 and CXCR2 signaling (Fig. 6a, b). The data were further confirmed by assessing ERK phosphorylation. It was found that CXCL8 induced robust phosphorylation but this signaling was impeded when the tyrosine residue was nitrated. Likewise, Y13F CXCL8 showed reduced activity when compared to wild type CXCL8, confirming the role of Tyr13 in receptor activation (Fig. 6c). Cell lines expressing CXCR1 or CXCR2 were used to characterize the activity of CXCL8 variants for β-arrestin recruitment. Nitration of Tyr13 in CXCL8 completely abrogated β-arrestin recruitment in CXCR1-expressing cells but only attenuated recruitment in CXCR2-expressing cells. This suggests that the tyrosine residue is more important for CXCR1 than for CXCR2 β-arrestin signaling. Furthermore, the importance of Tyr13 to elicit β-arrestin recruitment was highlighted in the Y13F CXCL8 (Fig. 7). These studies collectively suggest that Tyr13 in CXCL8 is important in G protein and β-arrestin signaling of CXCR1 and CXCR2 receptors and nitration attenuates CXCL8’s function albeit to a varying degree.

### Detection of nitrated CXCL8 in bronchoalveolar lavage

**Fig. 8:**
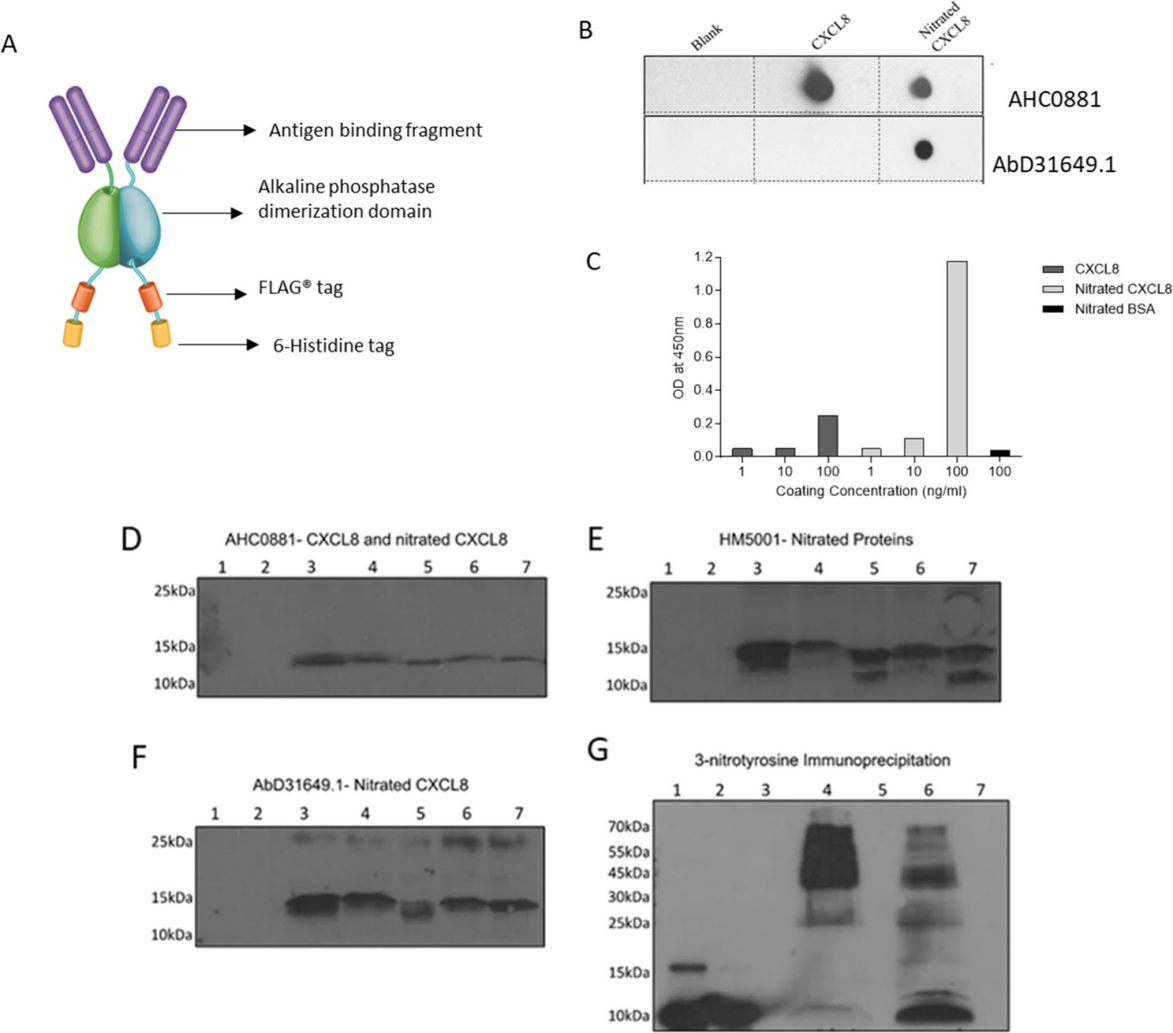
Naturally occurring nitrated CXCL8 in BAL samples from suspected-VAP patients. **a** Structure of HuCAL antibody, bivalent monoclonal antibody consisting of the antigen-binding fragment with two antigen recognition sites connected by an alkaline phosphatase dimerization domain with FLAG^®^ and Histidine 6 tags for detection. **b** Specificity of the antibody was tested via dot blot with wild type or nitrated CXCL8 detected with either AHC0881, which recognizes both variants of CXCL8 and HuCAL AbD31649.1, which recognizes only the nitrated variant and **c** ELISA. Nitrated BSA was used as a control to test cross-reactivity with a non-specific nitrated protein at the highest concentration. Detection was carried out using a biotinylated anti-histidine 6 secondary antibody (MCA1396B, Bio-Rad), streptavidin-HRP and TMB substrate, and the plate was read at 450 nm N = 3. **d** Identification of CXCL8, nitrated proteins and naturally occurring nitrated-CXCL8 using western blot analysis on BAL samples. **d**–**f** Lanes 1 and 2: healthy controls. Lanes 3–7: BAL samples from VAP patients. Gels were run in triplicate and the resulting membranes after protein transfer were probed with, **d** rabbit polyclonal anti-CXCL8 antibody (AHC0881), **e** mouse monoclonal anti-3-nitrotyrosine antibody (HM5001) and **f** HuCAL^®^ antibody (AbD31649.1). **g** Immunoprecipitation of BAL samples using 3-NT affinity columns and subsequently detecting for the presence of CXCL8. Lane 1: CXCL8, Lane 2: nitrated-CXCL8. Lane 3: blank. Lane 4: pulldown. Lane 5: blank. Lane 6: unbound fractions. Lane 7: blank

We generated recombinant monoclonal HuCAL^®^ antibodies [30] against our recombinant nitrated CXCL8 using phage display technology (**Fig. ****8****A**). The specificity of these antibodies was verified by dot blot where the HuCAL antibody detected nitrated CXCL8 but not wild type CXCL8 (Fig. 8b). The antibody generated showed specificity for nitrated CXCL8 over wild type CXCL8 at low concentrations (10–100 ng/ml) (Fig. 8c).

BAL fluid from patients with suspected-VAP were probed for the presence of nitrated CXCL8. This patient cohort was chosen, as it is well-established that CXCL8 is highly expressed and can be easily detected in the BAL samples from these patients [5, 18, 31]. BAL samples from healthy individuals were included as controls. The patients with suspected VAP were 7 male and 5 female with a median age 51 (range 22–69). Using antibodies that detect CXCL8 and 3-NT respectively (and by inference nitrated CXCL8); we showed that CXCL8 and nitrated proteins are elevated in diseased samples but not in the controls (Fig. 8, Supplementary Fig. 5). To confirm the presence of nitrated CXCL8, BAL samples were enriched using a commercially available 3-NT immunoprecipitation kit and CXCL8 was identified in the enriched fraction (Fig. 8g).

Importantly, HuCAL antibody showed the presence of nitrated CXCL8 in BAL samples from patients with pneumonia but not in controls (Fig. 8f and Supplementary Fig. 5). We found 75% (9/12) of the BAL samples from VAP patients showed the presence of nitrated CXCL8 in contrast to no detectable CXCL8 in healthy controls. Collectively, these results provided evidence for nitrated CXCL8 in these clinical samples. These data were further verified by mass spectrometry using the ISTAMPA workflow and nitrated CXCL8 was identified in 3 out of 12 samples (Supplementary Fig. 6). This discrepancy in detection of nitrated CXCL8 by HUCAL antibody and not by mass spectrometry could be attributed either to stability of nitration during processing of samples or assay sensitivity.

## Discussion

Neutrophil activation during inflammation must be tightly regulated to be robust to combat with pathogenic stimuli, without causing unnecessary damage to healthy tissue. The resolution of inflammation is now thought to be an active rather than a passive process. Peroxynitrite is a highly reactive species that is generated in situ by the reaction of the free radical superoxide and nitric oxide during sustained inflammation. The unstable ion potently modifies several residues and is a known feature of inflammation involving nitro-oxidative stress, which exacerbates injury by causing DNA double strand breaks [32] and lipid peroxidation [33]. In this study, we show that peroxynitrite-mediated nitration of CXCL8 alters its functionality with a potential to limit neutrophil-mediated inflammatory response.

We show that nitration of CXCL8 substantially impairs its ability to induce migration of neutrophils in vitro and in vivo*.* We further show that this functional loss can be attributed to impaired receptor signaling as evident from impaired calcium flux, β-arrestin recruitment and ERK phosphorylation. GAG binding was also impaired as inferred from SPR measurements. These findings were also translated to an in vivo murine intraperitoneal neutrophil recruitment model, which encompasses both receptor signaling and GAG binding. It was also found that introduction to the murine air pouch of an equimolar mixture of CXCL8 and nitrated CXCL8 did not antagonize the normal inflammatory response produced by wild type CXCL8. NMR studies indicated that nitration does not impact the structure of CXCL8 and so any changes in function are specifically due to nitration rather than to global structural changes.

CXCR1/2 receptor activation involves two distinct sites, an N-terminal domain (defined as Site-I) and a groove defined by extracellular loop/transmembrane helices (defined as Site-II). We have previously shown for CXCL8 that binding at these two distinct sites are not independent events, and that initial binding to the receptor N-domain at site-I results in structural changes that are coupled to binding at site-II and receptor activation [34]. Our recent structural and molecular dynamics studies show that Tyr13 plays an important role in the site-I reorganization and binding at site-II [34]. These structural insights indicate that Tyr13 nitration not only impacts binding at site-I but also binding at site II. Previous mutational studies have also shown that interactions of Tyr13 plays a more important role for CXCR1 binding and activation and that this position is less stringent for CXCR2 function. For instance, the Y13E mutant is impaired for both receptors but is 14-fold less active for CXCR1 compared to CXCR2 [35]. These and our data for the nitrated CXCL8 and Y13F mutant also suggest that more drastic substitution/mutation have a larger impact on CXCR1 than CXCR2 activity.

Tyr13, the nitration site, is in the N-loop and is far removed from the dimerization interface that consists of the b_1_-strand and the C-terminal helix. Our NMR data convincingly show the dimer interactions of nitrated CXCL8 is no different from native CXCL8. Therefore, heterodimerization and homodimerization will be equally favored (dimerization constant ~ 1 mM). In our in vitro functional assays, the active form is the monomer. Under conditions where nitrated CXCL8 could exist as a heterodimer, one of the two epitopes will be still available for antibody recognition. Our antibody detection studies cannot distinguish between the various oligomeric forms but only report whether the nitrated chemokine is present or not. Further, the CXCR2 activity of the heterodimer will be similar to the homodimer as only one monomer of the dimer engages the receptor with the other monomer pointed away from the receptor.

The majority of studies assessing the involvement of chemokines in disease assume that all of the chemokine is fully functional. Most current detection methods do not differentiate between wild type and modified forms of a chemokine. Our strategy of generating an antibody that shows specificity for nitrated CXCL8 was crucial for the detection of nitrated CXCL8 in clinical samples. We found 75% (9/12) of the BAL samples from suspected-VAP patients showed the presence of nitrated CXCL8 in contrast to no detectable CXCL8 or nitrated CXCL8 in healthy controls. These data confirm that CXCL8 can be subjected to tyrosine nitration during inflammatory processes without CXCL8 degradation. To our knowledge, this is the first demonstration of the presence of nitrated CXCL8 in clinical samples.

Chemokine function is regulated at many levels, including the ability to exist as monomers and dimers [9], binding to GAGs [36] and binding to one or more receptors that trigger activation of G protein and β-arrestin signaling pathways [29, 37]. Our current study shows altered function of a chemically modified chemokine, suggesting an additional level of regulation. In some instances, nitration can prevent detection of chemokines by antibodies [13, 38], potentially limiting the biological relevance of some immunochemical/proteomic biomarker tests. While measurements of CXCL8 have been correlated with inflammatory diseases, this could be the reason that none has been translated into a clinical benchmark. To better understand the quantity and patterns in expression of nitrated and wild type chemokines and their relationships with disease states, there is a need to develop techniques for unambiguous detection of nitrated chemokines separately from their wild type counterparts. This can also be extrapolated to other forms of modified chemokines, such as truncated and citrullinated variants. Teasing out and quantifying the relative amounts of all chemokine forms, if the functionality and circumstances of production of each is understood, would allow for complete and accurate assessment of post-translational modifications regulating chemokine function during inflammation. Better understanding of natural chemokine regulation could help the development of therapeutics targeting the chemokine system, and their use as clinical biomarkers. In conclusion, this is the first demonstration that CXCL8 can be nitrated in human inflammatory conditions (e.g., suspected VAP). Furthermore, the nitration renders it biologically inactive.

## Supplementary Information

Below is the link to the electronic supplementary material.

Supplementary file1 (DOCX 503 KB)

## Data Availability

All data are available in the main text or the supplementary materials.

## References

[1] Phillipson M, Kubes P (2019). The healing power of neutrophils. Trends Immunol.

[2] Lacy P (2006). Mechanisms of degranulation in neutrophils. Allergy Asthma Clin Immunol.

[3] Ciz M, Lojek A (2013). Modulation of neutrophil oxidative burst via histamine receptors. Br J Pharmacol.

[4] Klebanoff SJ (2013). Myeloperoxidase: a front-line defender against phagocytosed microorganisms. J Leukoc Biol.

[5] Cambier S (2022). Atypical response to bacterial coinfection and persistent neutrophilic bronchoalveolar inflammation distinguish critical COVID-19 from influenza. JCI Insight.

[6] Martins-Green M, Petreaca M, Wang L (2013). Chemokines and their receptors are key players in the orchestra that regulates wound healing. Adv Wound Care (New Rochelle).

[7] Matsushima K (1988). Molecular cloning of a human monocyte-derived neutrophil chemotactic factor (MDNCF) and the induction of MDNCF mRNA by interleukin 1 and tumor necrosis factor. J Exp Med.

[8] Matsushima K, Yang D, Oppenheim JJ (2022). Interleukin-8: an evolving chemokine. Cytokine.

[9] Das ST (2010). Monomeric and dimeric CXCL8 are both essential for in vivo neutrophil recruitment. PLoS ONE.

[10] Joseph PRB, Sawant KV, Rajarathnam K (2017). Heparin-bound chemokine CXCL8 monomer and dimer are impaired for CXCR1 and CXCR2 activation: implications for gradients and neutrophil trafficking. Open Biol.

[11] Lux M (2019). The atypical chemokine receptor 2 limits progressive fibrosis after acute ischemic kidney injury. Am J Pathol.

[12] Metzemaekers M (2021). From ELISA to immunosorbent tandem mass spectrometry proteoform analysis: the example of CXCL8/Interleukin-8. Front Immunol.

[13] Barker CE (2017). CCL2 nitration is a negative regulator of chemokine-mediated inflammation. Sci Rep.

[14] Sato E (1999). Effects of reactive oxygen and nitrogen metabolites on RANTES- and IL-5-induced eosinophil chemotactic activity in vitro. Am J Pathol.

[15] Janssens R (2016). Natural nitration of CXCL12 reduces its signaling capacity and chemotactic activity in vitro and abrogates intra-articular lymphocyte recruitment in vivo. Oncotarget.

[16] Szabo C, Ischiropoulos H, Radi R (2007). Peroxynitrite: biochemistry, pathophysiology and development of therapeutics. Nat Rev Drug Discov.

[17] Beckman JS (1990). Apparent hydroxyl radical production by peroxynitrite: implications for endothelial injury from nitric oxide and superoxide. Proc Natl Acad Sci USA.

[18] Hellyer TP (2015). Diagnostic accuracy of pulmonary host inflammatory mediators in the exclusion of ventilator-acquired pneumonia. Thorax.

[19] Ades EW (1992). HMEC-1: establishment of an immortalized human microvascular endothelial cell line. J Investig Dermatol.

[20] Martinez-Burgo B (2019). A C-terminal CXCL8 peptide based on chemokine-glycosaminoglycan interactions reduces neutrophil adhesion and migration during inflammation. Immunology.

[21] Sepuru KM, Rajarathnam K (2018). Distinct differences in structural states of conserved histidines in two related proteins: NMR studies of the chemokines CXCL1 and CXCL8 in the free form and macromolecular complexes. Biochemistry.

[22] Hol J, Wilhelmsen L, Haraldsen G (2010). The murine IL-8 homologues KC, MIP-2, and LIX are found in endothelial cytoplasmic granules but not in Weibel-Palade bodies. J Leukoc Biol.

[23] Gangavarapu P (2012). The monomer-dimer equilibrium and glycosaminoglycan interactions of chemokine CXCL8 regulate tissue-specific neutrophil recruitment. J Leukoc Biol.

[24] Joseph PR (2015). Solution NMR characterization of chemokine CXCL8/IL-8 monomer and dimer binding to glycosaminoglycans: structural plasticity mediates differential binding interactions. Biochem J.

[25] Joseph PR, Rajarathnam K (2015). Solution NMR characterization of WT CXCL8 monomer and dimer binding to CXCR1 N-terminal domain. Protein Sci.

[26] Sepuru KM, Rajarathnam K (2019). Structural basis of chemokine interactions with heparan sulfate, chondroitin sulfate, and dermatan sulfate. J Biol Chem.

[27] Metzemaekers M, Gouwy M, Proost P (2020). Neutrophil chemoattractant receptors in health and disease: double-edged swords. Cell Mol Immunol.

[28] Rajarathnam K (2019). How do chemokines navigate neutrophils to the target site: dissecting the structural mechanisms and signaling pathways. Cell Signal.

[29] Nasser MW (2009). Differential activation and regulation of CXCR1 and CXCR2 by CXCL8 monomer and dimer. J Immunol.

[30] Knappik A (2000). Fully synthetic human combinatorial antibody libraries (HuCAL) based on modular consensus frameworks and CDRs randomized with trinucleotides. J Mol Biol.

[31] Conway Morris A (2010). Diagnostic importance of pulmonary interleukin-1beta and interleukin-8 in ventilator-associated pneumonia. Thorax.

[32] Epe B (1996). DNA damage by peroxynitrite characterized with DNA repair enzymes. Nucleic Acids Res.

[33] Radi R (1991). Peroxynitrite-induced membrane lipid peroxidation: the cytotoxic potential of superoxide and nitric oxide. Arch Biochem Biophys.

[34] Sepuru KM (2020). Long-range coupled motions underlie ligand recognition by a chemokine receptor. iScience.

[35] Lowman HB (1996). Exchanging interleukin-8 and melanoma growth-stimulating activity receptor binding specificities. J Biol Chem.

[36] Ali S (2010). Therapy with nonglycosaminoglycan-binding mutant CCL7: a novel strategy to limit allograft inflammation. Am J Transplant.

[37] Joseph PR (2013). Dynamic conformational switching in the chemokine ligand is essential for G-protein-coupled receptor activation. Biochem J.

[38] Molon B (2011). Chemokine nitration prevents intratumoral infiltration of antigen-specific T cells. J Exp Med.

