# Supplementary material for: Nitration of chemokine CXCL8 acts as a natural mechanism to limit acute inflammation
Source: Cell Mol Life Sci. 2023 Jan 9;80(1):35. doi: 10.1007/s00018-022-04663-x (PMC9829591; doi:10.1007/s00018-022-04663-x)
Supplement: Supplementary file 1 — Supplementary file1 (DOCX 503 KB) [file 18_2022_4663_MOESM1_ESM.docx]

**Supplementary data**:


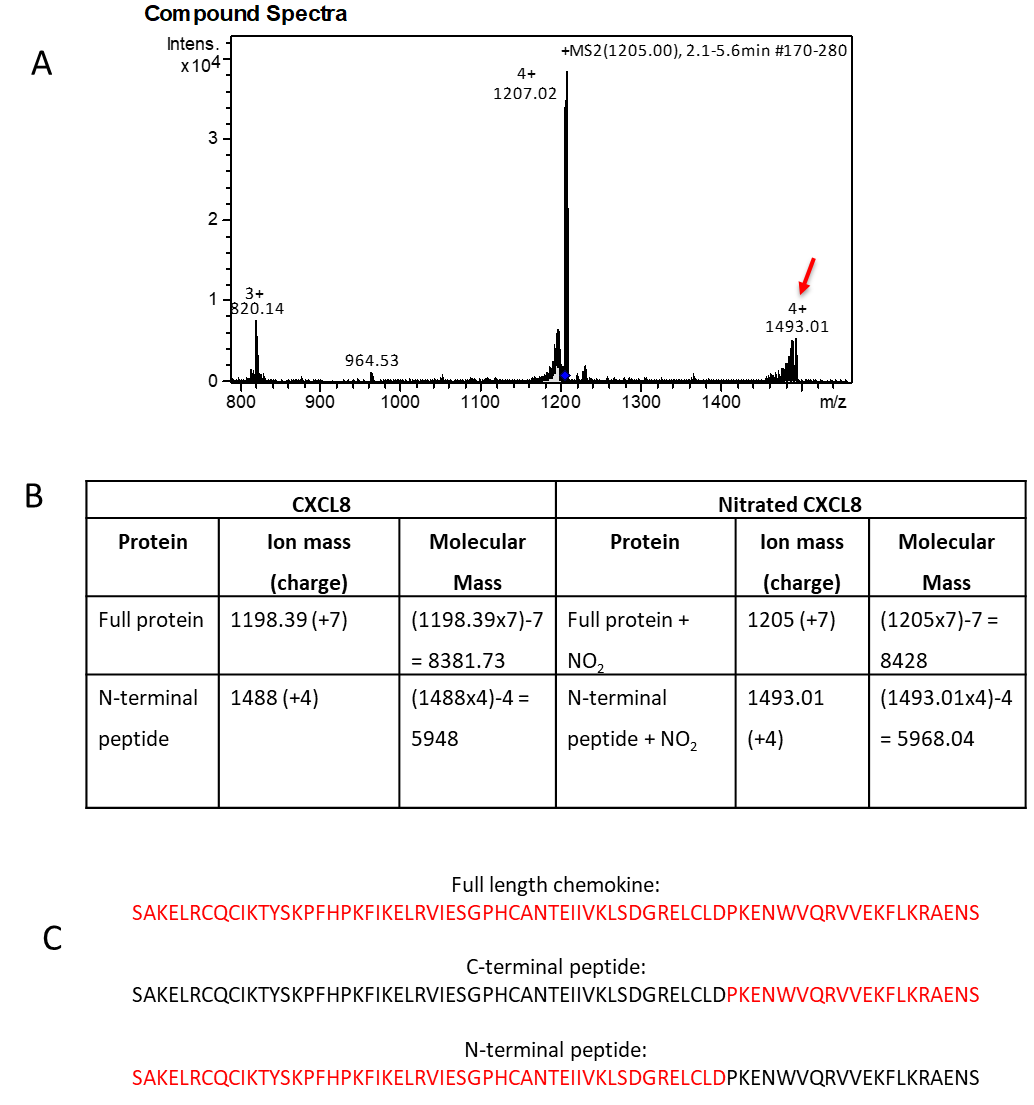


**Supplementary Figure 1.** **MS2 analysis of nitrated CXCL8.** A) Shows the compound spectra for MS2 analysis of nitrated CXCL8. Nitrated CXCL8 was manually injected at and analysed by ion trap mass spectrometry. The most abundant ion was the 1205 peak, which corresponds to a +7 charged ion of the full-length chemokine with one NO_2_ group added (+45Da). This ion was selected and held within the ion trap, then fragmented to break the D-P bond, thus cleaving the chemokine into a C-terminal peptide and an N-terminal peptide. A triple charged ion of 820.14 corresponds to a peptide with no additional NO_2_ groups, whereas the quadruple charged ion of 1493.01 (red arrow) is consistent with a peptide plus one NO_2_ group – therefore, incubation of CXCL8 with peroxynitrite results in one NO_2_ group being added to an amino acid within the N-terminal section of the chemokine. B) Shows the calculation method derived from the equation “peak readout = (m+z/z)” to determine the molecular mass of each ion. C) The CXCL8 amino acid sequence with the relative amino acids highlighted in red for each peak analysed.

**
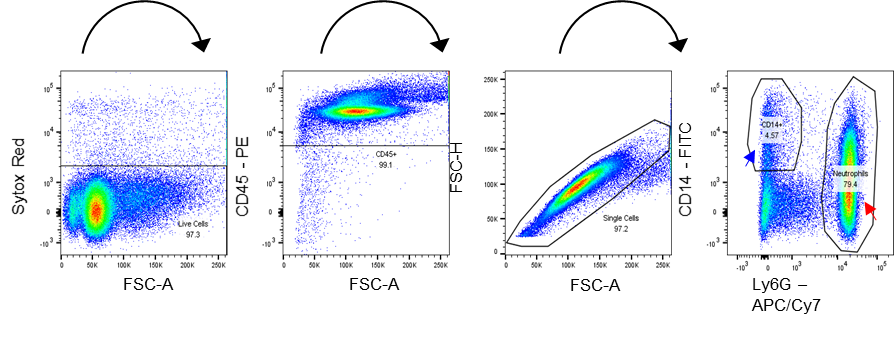
**

**Supplementary Figure 2.** **Gating strategy for neutrophil identification in murine intraperitoneal recruitment.** Sequential gating strategy used to characterise the neutrophils recruited into the murine peritoneal cavity.

**
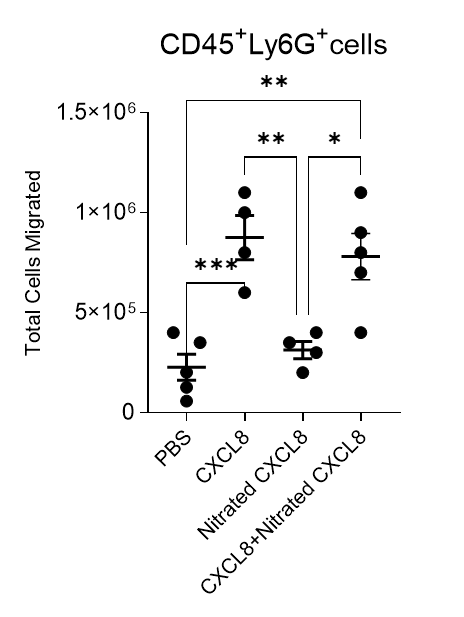
**

**Supplementary Figure 3. Nitrated CXCL8 does not antagonise the effects of CXCL8 in vivo.** Neutrophil infiltration into murine air pouch in response to wild type CXCL8, nitrated CXCL8 or both together was examined. Total neutrophil migration into air pouches 4 hours after intrapouch administration of PBS + 0.5% CMC, 1µg CXCL8 (in PBS + 0.5% CMC), 1µg Nitrated CXCL8 (in PBS + 0.5% CMC), or 1µg of each CXCL8 and Nitrated CXCL8 together, was determined. Cells were counted by using a TALI automatic cell counter, then stained for CD45, CD14 and Ly6G. Amounts of each cell type (gated at CD45+ Ly6G+ or CD45+ CD14+) were recorded using a FACS Canto III flow cytometer and analysed using FlowJo V10 software. Statistical analysis by ANOVA with Tukey’s post-test. Each symbol represents an animal.

**
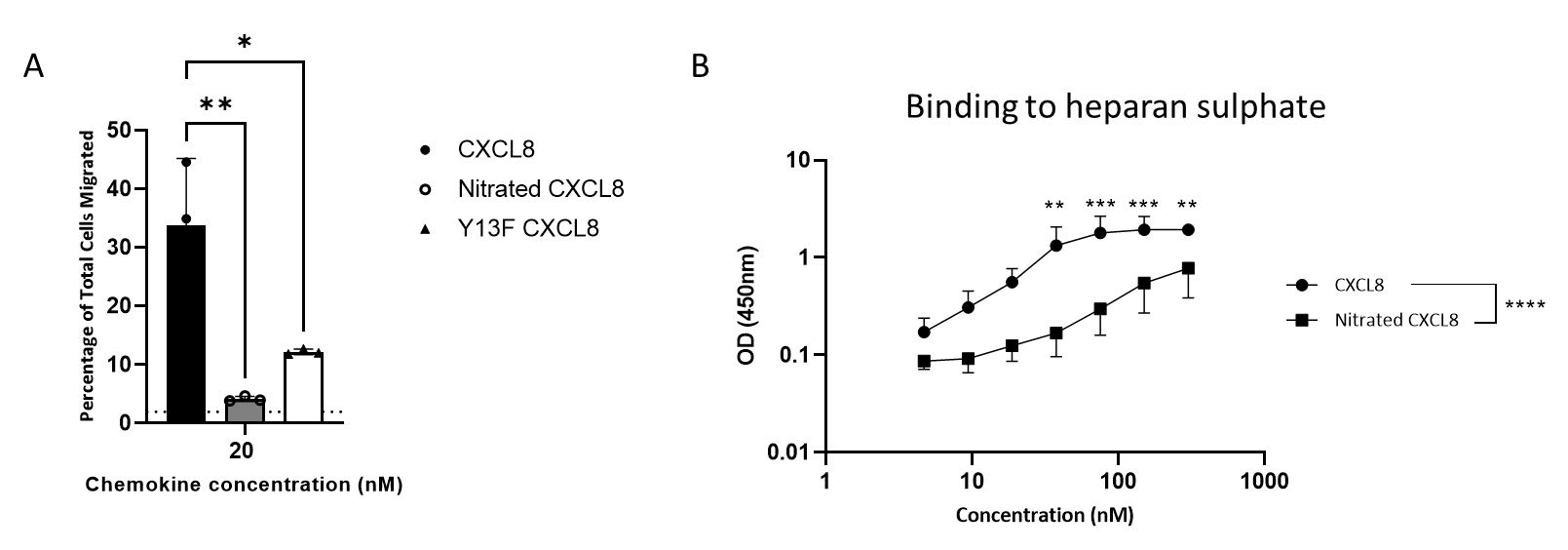
**

**Supplementary Figure 4: A) Ability of wild type CXCL8, nitrated CXCL8 and Y13F CXCL8 to induce neutrophil trans-enodthelial migration *in vitro.*** Trans-endothelial chemotaxis assay, showing percentage of total neutrophils that migrated through the confluent layer of HMEC-1. Data shown is representative of, N=2 experimental replicates (using neutrophils isolated from different blood donors), each with n=3 technical replicates. Dotted line indicates background migration in absence of chemokine. Statistical analysis was performed using a One Way ANOVA with Tukey’s post-test.

B**) Binding of the wild type and nitrated CXCL8 to heparan sulphate**: Heparan sulfate (25μg/ml) was coated overnight at room temperature on a GAG binding plate. Serial dilutions of CXCL8 variants (4.6875nM- 300nM) were added in technical triplicates and incubated for 2 hours at room temperature. Subsequently, bound CXCL8 were detected using anti-CXCL8 (Invitrogen, AHC0881) which recognizes both variants with equivalent efficiency and polyclonal goat-anti rabbit HRP antibody (Sigma, A6154). The peroxidase activity was quantified using 3,3′-5,5′-tetramethylbenzidine (TMB) substrate conversion and the absorbance was read at 450 nm. N=3.


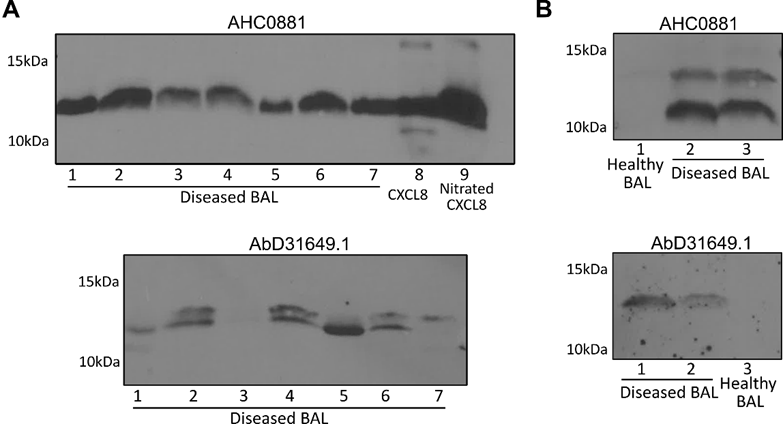


**Supplementary Figure 5**. **Validation of CXCL8 and naturally occurring nitrated CXCL8 in bronchoalveolar lavage samples from patients with ventilator-associated pneumonia.** Additional screening of broncheoalveolar lavage sample from patients with ventilator-associated pneumonia to validate the presence of CXCL8 and nitrated CXCL8 in biological samples. Rabbit polyclonal anti-CXCL8 antibody (AHC0881), and HuCAL® antibody (AbD31649.1).

**
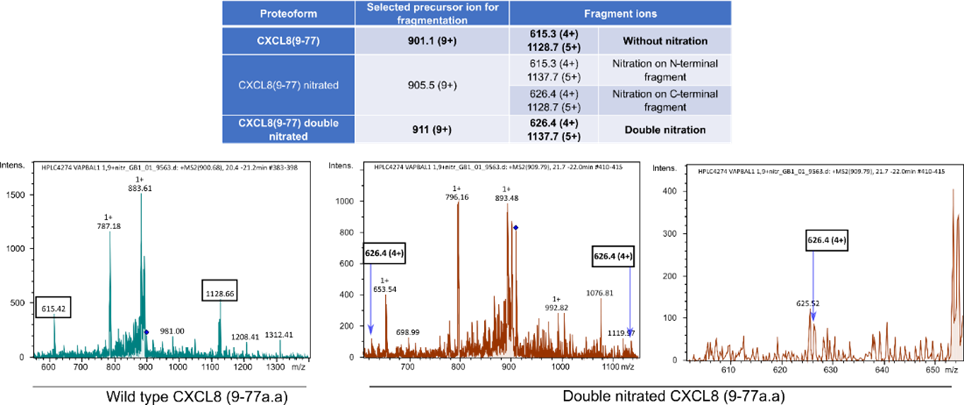
**

**Supplementary Figure 6**. **Mass spectrometry detection of nitrated CXCL8.** Presence of nitrated CXCL8 detected by HuCAL® antibody was validated in the 3/12 samples by nano HPLC-MS/MS using ISTAMPA methodology.

**Supplementary methods:**

**Cell recruitment to murine air pouches:**  Eight-week-old female BALB/c mice (Charles River) were used for generation of air pouches in full compliance with U.K. Home Office regulations for animal experimentation. Briefly, air pouches were induced by injecting 3 ml of sterile air s.c. into the back of each animal followed by 1 ml of air on three further occasions (days 2, 4, and 5 respectively); this produced stable fluid-filled pouches. On day 6, each pouch was injected with 1 ml of PBS containing either 1 µg of CXCL, nitrated CXCL8 or a combination of both. Identical age-matched control mice were injected with PBS alone. Six hour later, recruited cells were recovered by gently lavaging the pouch with 1 ml of PBS containing 1 mM EDTA. The exudates were centrifuged at 1000 g for 5 min and the supernatants removed. The cell pellets were resuspended in 1 ml of PBS for counting and assessment of viability.

**Glycosaminoglycan binding assay**: Heparan sulfate/heparin (25μg/ml) was coated overnight at room temperature on a GAG binding plate (BD Biosciences). Serial dilutions of CXCL8 variants (4.6875nM- 300nM) were added in technical triplicates and incubated for 2 hours at room temperature. Subsequently, bound CXCL8 were detected using anti-CXCL8 (Invitrogen, AHC0881) which recognises both variants with equivalent efficiency and polyclonal goat-anti rabbit HRP antibody (Sigma, A6154). The peroxidase activity was quantified using 3,3′-5,5′-tetramethylbenzidine (TMB) substrate conversion and the absorbance was read at 450 nm.
